# Partially Overlapping Primer-Based PCR for Genome Walking

**DOI:** 10.1371/journal.pone.0120139

**Published:** 2015-03-26

**Authors:** Haixing Li, Dongqin Ding, Yusheng Cao, Bo Yu, Liang Guo, Xiaohua Liu

**Affiliations:** Sino-German Joint Research Institute, Nanchang University, Nanchang, Jiangxi, People’s Republic of China; University of Bonn, Institut of experimental hematology and transfusion medicine, GERMANY

## Abstract

Current genome walking methods are cumbersome to perform and can result in non-specific products. Here, we demonstrate the use of partially overlapping primer-based PCR (POP-PCR), a direct genome walking technique for the isolation of unknown flanking regions. This method exploits the partially overlapping characteristic at the 3’ ends of a set of POP primers (walking primers), which guarantees that the POP primer only anneals to the POP site of the preceding PCR product at relatively low temperatures. POP primer adaptation priming at the genomic DNA/POP site occurs only once due to one low-/reduced-stringency cycle in each nested PCR, resulting in the synthesis of a pool of single-stranded DNA molecules. Of this pool, the target single-stranded DNA is replicated to the double-stranded form bound by the specific primer and the POP primer in the subsequent high-stringency cycle due to the presence of the specific primer-binding site. The non-target single stranded DNA does not become double stranded due to the absence of a binding site for any of the primers. Therefore, the POP-PCR enriches target DNA while suppressing non-target products. We successfully used POP-PCR to retrieve flanking regions bordering the *gadA* locus in *Lactobacillus brevis* NCL912, *malQ* in *Pichia pastoris* GS115, the human *aldolase A* gene, and *hyg* in rice.

## Introduction

Molecular biological research frequently requires DNA isolation from an unknown region bordering a known sequence [1–3]. Conventional approaches for isolating flanking regions from known genomic sequences are generally tedious and labor-intensive, because genomic DNA library construction and screening are time-consuming with a heavy workload. PCR-based genome walking strategies facilitate the rapid isolation of unknown flanking DNA regions. These methods can differ substantially in complexity and effectiveness, but essentially fall into three types [4]: (I) inverse PCR [5]; (II) restriction-ligation/tailing mediated PCR, including adaptor/cassette/linker ligation PCR [6–10], restriction site extension PCR [11], and panhandle PCR [12]; and (III) random primed PCR, including self-formed adaptor PCR (SFA-PCR) [13], thermal asymmetric interlaced PCR (TAIL-PCR) [1], SiteFinding-PCR [14], and single primer-mediated TA-cloning PCR [15].

For inverse PCR, genomic DNA is digested with a restriction enzyme, and then self-circularized to form a circle containing the original unknown upstream and downstream regions ligated together. The circularized DNA serves as a template for PCR using two specific primers oriented in the reverse direction [5]. The methods in the aforementioned type II PCR-based genome walking strategies involve restriction of genomic DNA and subsequent ligation/tailing-mediated PCR. PCR amplification is then conducted using a nested specific primer and an adaptor/tail primer [11,12,16–18]. In type III methods, SFA-PCR uses a walking primer (both ends are specific and the middle region is degenerate) to place known DNA on the unknown side of the sequence of interest via loop-back extension-mediated generation of a molecule that is shaped like a pan with a handle. This molecule serves as a template for PCR using specific primer(s) [13]. TAIL-PCR utilizes one low-stringency PCR cycle to facilitate priming of a shorter arbitrary degenerate primer. Then, differential amplification during PCR is achieved by repeating the combination of one low-stringency cycle and two high-stringency cycles, which favors amplification of the desired PCR products [1]. The basic principle of the other randomly primed PCR is similar to that of TAIL-PCR except that there is no strategy for eliminating non-target products during PCR, and therefore, subsequent clone screening is inevitable with this method [14,15]. These genome walking strategies either require additional manipulations, or are prone to high background levels. Several commercial kits for genome walking are available based on the above strategies (for details, see the review article [3]).

Here, we present a new genome walking technology, termed partially overlapping primer-based PCR (POP-PCR), which isolates unknown flanking DNA regions without the need of prior DNA manipulation or further operating procedures after PCR. The partially overlapping characteristic at the 3’ ends of a set of relative long POP primers is designed to remove non-target products while effectively enriching the target DNA. The feasibility of the developed method was verified by retrieving fragments of interest from the genomic DNA of *L*. *brevis* NCL912, *P*. *pastoris* GS115, humans, and rice.

## Materials and Methods

### Genomic DNA isolation and purification

The genomic DNA of NCL912 and human blood were extracted by the methods previously described [19,20]. Genomic DNA was extracted from *P*. *pastoris* GS115 cells, and purified using the Dr. GenTLE (from Yeast) High Recovery Kit (TaKaRa, Dalian, China) according to the manufacturer’s instructions.

### Primers

A POP primer set consists of three POP primers [POP-P (primary PCR), POP-S (secondary PCR), POP-T (tertiary PCR)], which are completely arbitrary and have 10 base pair (bp) identical 3’ ends and 15 bp heterologous 5’ ends. This partially overlapping design guarantees that the POP primers only anneal to each other’s complementary site at relatively low temperatures. The POP primers should simultaneously meet the following rules. Four bases A, T, G, and C are evenly distributed in each primer without any degeneracy or base modification, and the G+C content of the 10 bp overlap is between 50% and 60%. Each primer had a relatively high melting temperature (65–70°C) according to Mazars *et al*. [21], and should not self-anneal to form internal hairpins and loops. Primer dimers should be avoided in the same POP primer set. In this study, four POP primer sets (POPx-P, POPx-S, POPx-T [x = 1, 2, 3, or 4]) were designed for parallel DNA walking.

Using the DNA sequences of glutamate decarboxylase gene (*gadA*) locus (GenBank accession number JX074764) of *L*. *brevis* NCL912 [19], amylomaltase gene (*malQ*) (AM946981.2) integrated in the genome of *P*. *pastoris* GS115 [22], human aldolase A gene (ALDOA) (AC_000148.1), and hygromycin gene (*hyg*) (KF206149.1) integrated in the genome of rice, two sets of gene-specific primers in nested positions were selected from each gene (locus). Each specific primer had a similar melting temperature with its paired POP primer. Any specific POP primer pair should avoid forming dimers. Other rules in designing specific primers were generally the same as those for normal PCR. The primers used in this study are listed in Table 1.

**Table 1:** Primers used in this study.

Primer set^1^	Primary PCR	Secondary PCR	Tertiary PCR	Gene (locus) walked and direction
POP1	AGTCAGCGTCCAGGT*AGTCAGTCTC*	TCAGGTCCAAGGTCA*AGTCAGTCTC*	CTCAGCGTGTTCGTC*AGTCAGTCTC*	
POP2	CAGTCAGTCTCAGGT*CGTCTCCAGT*	AGCAGGTCAGTTACA*CGTCTCCAGT*	TCAGTCAGTCAGTTGCGTCTCCAGT	
POP3	CGCTTCAGATGGTAC*AGTGCAGTCA*	ACACGATCCCAAGGT*AGTGCAGTCA*	GTTACTCAGGTCCCA*AGTGCAGTCA*	
POP4	GCCTTGAACTGGACC*TGATCGACTG*	CATGACCGTGCTGAG*TGATCGACTG*	TGGACTGTGCTACCT*TGATCGACTG*	
5’-gadA	CATTTCCATAGGTTGCTCCAAGGTC	ACGTCATCTCAGTTGTTAGCCAACC	AGCCGGTTTGCTTTCAAATGATTCT	5’ region of *gadA* locus
3’-gadA	TGCGGATACTGATAACAAGACGACA	GGATTGAGAAAGAACGTACGGGTGA	TCCTGCATATCGGTAACGCCCAATC	3’ region of *gadA* locus
5’-ALDOA	AAATGCTGCAGCCTCCCTCTCACCC	AATACCAGAAATGTGCCCTCCCGTG	TGAGCTGGCAGGTTGTAGTCTCTGT	5’ region of ALDOA
3’-ALDOA	CCCTCGGACGATTGGACCTAGCTTG	GGTCTAACGGTGCCTCTCAGCCTCT	TCTGCCCTTCCCCATGGACGTAAGT	3’ region of ALDOA
5’-malQ	CTTCCTGGGTAAGCGTCAGCGTGTG	CAGCTTCGTCGGTAGATTGAACGCT	GGTGGTCAGCAGCCAGCTATATTCG	5’ region of *malQ*
3’-malQ	CGTCATCGCTGTATGGTGATTGGTG	CGGTGTTTACTCCTACAAAGTGCTC	GCTACATTGCCGACAGTAACAGTGC	3’ region of *malQ*
5’-hyg	CGGCAATTTCGATGATGCAGCTTGG	CGGGACTGTCGGGCGTACACAAATC	GACCGATGGCTGTGTAGAAGTACTC	5’ region of *hyg*
3’-hyg	AACTCCCCAATGTCAAGCACTTCCG	GAAACCATCGGCGCAGCTATTTACC	GAAAGCACGAGATTCTTCGCCCTCC	3’ region of *hyg*

^1^Each set of specific primers was respectively paired with four sets of POP primers to PCR; meanwhile a POP primer and a specific primer in the same column were matched for a corresponding round of PCR. Each POP primer set (the same row) consists of three POP primers having 10 bp overlap at the 3’ ends (italic).

### PCR procedure

Three rounds of PCR (primary, secondary, tertiary) were performed at each walking process using the product of the previous PCR as a template for the next PCR. The primary PCR reaction mixture was 1× LA PCR buffer II (Mg^2+^ plus) containing 0.4 mM dNTP, 0.2 μM of each primer, genomic DNA plate (10–100 ng for microbes and 100–1000 ng for human or rice), and 2.5 U TaKaRa LA Taq HS in a 50 μL reaction volume. The secondary PCR/tertiary PCR mixture consisted of 1× LA PCR buffer II (Mg^2+^ plus) containing 0.4 mM dNTP, 0.2 μM of each primer, 1 μL of the previous round of PCR product, and 2.5 U TaKaRa LA Taq HS in a 50 μL reaction volume.

Each round of PCR constituted three annealing stages: stage 1, five high-stringency (65°C) cycles; stage 2, one low- (25°C)/reduced-stringency (50°C) cycle; and stage 3, thirty high-stringency (65°C) cycles. Reaction profiles for the three rounds of PCR are listed in S1 Table.

### DNA manipulation and sequencing

PCR products were purified with the Agarose Gel DNA Purification Kit Ver.2.0 (TaKaRa), and were directly sequenced by Sangon Biotech Co., Ltd. (Shanghai, China) or ligated into pMD18-T Vector or pMD19-T Simple Vector (TaKaRa). Then, the recombinant plasmids were transformed into *E*. *coli* DH5α cells according to TaKaRa’s guidelines, and were sequenced by Sangon Biotech Co., Ltd.

## Results and Discussion

**Fig 1:**
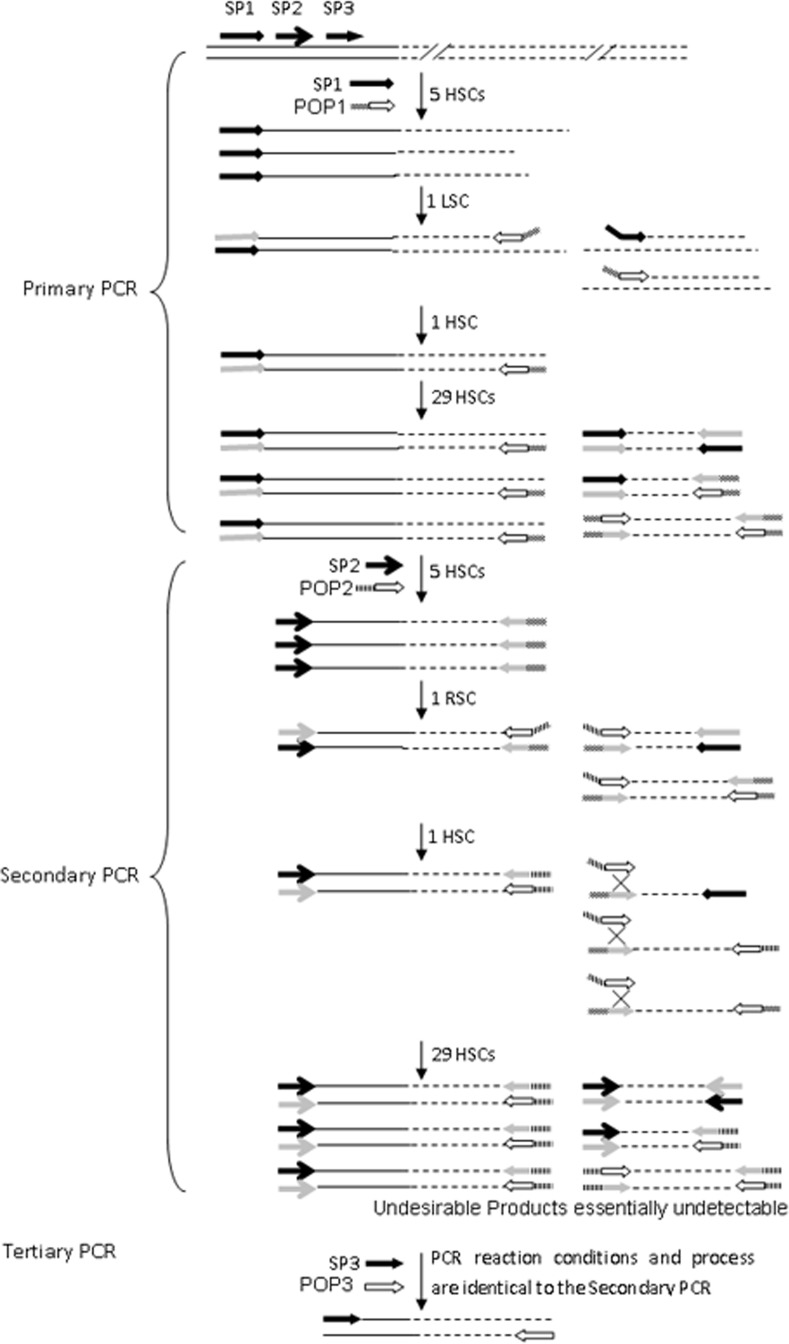
Overview of primer partially overlapping-based PCR. The first five high-stringency cycles (HSC) of each PCR are to increase copies of the single-stranded DNA of interest. The one low-stringency cycle (LSC) of primary PCR facilitates POP-P annealing to the target DNA and extension towards SP1. The one reduced-stringency cycle (RSC) of secondary PCR allowed POP-S to bind to the POP-P annealing site. A double-stranded target molecule was synthesized in the first HSC following LSC/RSC, and served as the template for the remaining HSCs; non-specific amplification was inhibited because the double-stranded form could not be obtained from a non-target single strand. Solid lines: the known sequence; dotted lines: the unknown sequence; thick black arrows with different heads: nested, specific primers; hollow arrows with different tails: POP primers; gray arrows: primers complementary sequences.

The key to the POP-PCR method is to utilize a set of POP primers having a 10 bp overlap at the 3’ ends, which only anneal to each other’s complementary site at a relatively low temperature. An overview of the procedure is presented in Fig. 1. Three rounds of nested PCR were successively performed at each genome walking. In each nested PCR, specific priming within the known sequence in the first five high-stringency cycles increased the copy number of single-stranded DNA (ssDNA) of interest. The one low-/reduced-stringency cycle allowed the POP primer to anneal to the genomic DNA/POP primer site of the preceding PCR product only once, thus creating a pool of nascent ssDNAs (consisting of target and non-target ssDNAs) with the POP primer sequence at the 5’ end. In the subsequent high-stringency cycle, the specific primer annealed to the specific binding site within the target ssDNA and extended towards the POP primer site, producing a double-stranded target molecule bound by the two primers. This double-stranded molecule was exponentially amplified in the remaining high-stringency cycles. However, the non-target ssDNAs could not be converted into double-stranded form due to the lack of a perfect binding site for any primers. Therefore, amplification of the non-target product was suppressed. Finally, the target molecule became the major product.

Three types of non-target products were formed in the POP-PCR: (I) primed by specific primer alone; (II) primed by specific and POP primers; and (III) primed by POP primer alone. Types I and II undesired PCR products were easily diluted in the subsequent PCR using a specific internally nested primer. The biggest challenge in PCR-based genome walking is eliminating type III non-target products [1]. Here, a POP primer set was designed to remove this kind of non-target products. The partially overlapping characteristic of a POP primer set made the latter POP primer anneal to the former POP primer site at only the one reduced-stringency cycle for initiation of a new type III ssDNA synthesis. However, the 3’ end of newly produced non-target ssDNA was still the complementary sequence of the former POP primer, which could not hybridize to the latter POP primer in the subsequent high-stringency cycle, resulting in no further amplification of type III products. Therefore, type III nonspecific products were easily eliminated by altering POP primers in the subsequent PCR. The POP-PCR strategy favors amplification of desired specific products and suppresses amplification of nonspecific products.

It should be mentioned that a POP primer has a high melting temperature similar to its paired specific primer. In the high-stringency PCR cycles, the role of a POP primer is actually equivalent to that of a specific primer. This characteristic contributes not only to the specificity of POP-PCR, but also to its efficiency. In order to guarantee that only one low-/reduced-stringency cycle occurs in each PCR, the Hot Start Long PCR, a modified form of PCR that avoids nonspecific amplification during PCR reaction solution preparation by inactivating the Taq polymerase at a lower temperature, should be utilized.

**Fig 2:**
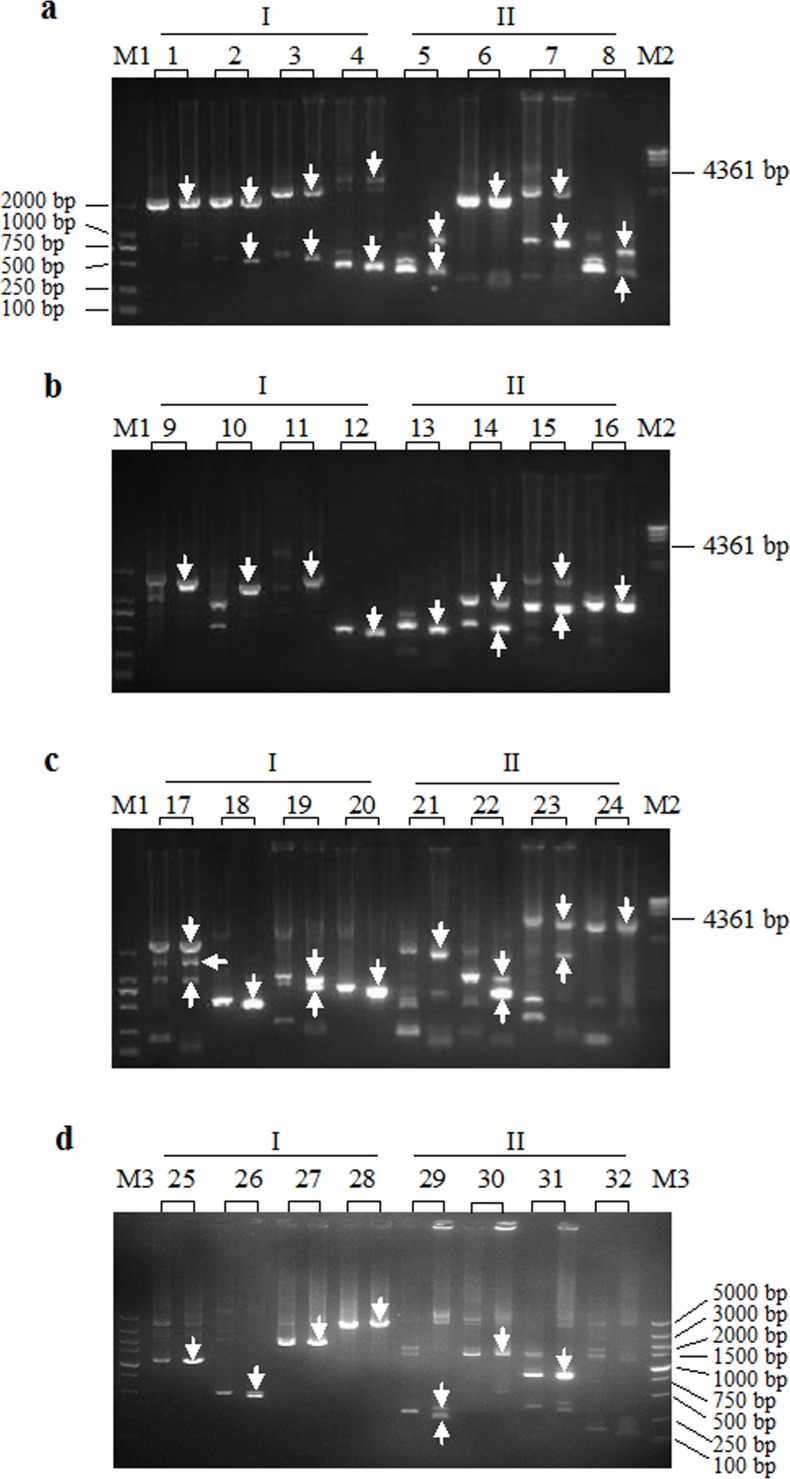
Chromosome walking of the *gadA* locus of *Lactobacillus brevis* NCL912 (a), human aldolase A gene (b), *malQ* of *Pichia pastoris* GS115 (c), and *hyg* of rice (d). I: walking into 5’ regions of the genes (locus); II: walking into 3’ regions of the genes (locus). Each walking experiment contained four sets of PCRs that respectively utilized the four POP primer sets, POP1, POP2, POP3, and POP4, paired with a specific primer set. For each set of PCRs, only the results of secondary PCR (left lane) and tertiary (right lane) PCR are presented. White arrows indicate target bands. M1: DL2000 DNA marker. M2: λ-Hind III digest DNA Marker. M3: DL5000 DNA marker.

To demonstrate the feasibility of the method in retrieving unknown sequences around a known sequence, we employed this technique to identify target sequences bordering the *gadA* locus in *L*. *brevis* NCL912 [19], *malQ* in *P*. *pastoris* GS115 [22], the human aldolase A gene (ALDOA), and *hyg* in rice (Table 1). Eight sets of specific primers (two for each gene [locus]) were respectively paired with the four POP primer sets, resulting in eight DNA walking reactions (8×4 = 32 sets of PCR reactions). Clear main DNA band(s) appeared in each walking reaction (31 of 32 sets of PCR present positive results) (Fig. 2). In the first round of nested PCR, the combination of one low-stringency and a relatively long POPx-P primer should create annealing site(s) adapted for the POPx-P within the unknown target sequence bordering the known sequence. We believe that at least one POP primer set can generate a positive product if the four POP primer sets are simultaneously performed.

The longest amplified fragments in each walking experiment ranged from 1.3 kb to 3.5 kb in size (average was up to 2.2 kb). The products were sequenced, and verification that the products originated in a region that completely overlapped the end of the known sequence, was performed. Then, the obtained sequences were assembled with the corresponding known sequence. We amplified each assembled fragment using two specific primers selected from both ends, and sequence analysis indicated that the fragment contained the known and retrieved sequences. In addition, DNA homology searches against GenBank showed that the obtained sequences were correct (data not shown). The new sequences obtained from *L*. *brevis* NCL912 were deposited in GenBank (accession numbers KJ413011 and KJ413012).

POP-PCR cycling is usually unnecessary in tertiary PCR. In almost all cases, the distinct specific product band(s) were produced from the secondary PCR. Product specificity was easily confirmed by stepwise changes in the sizes of PCR products that corresponded to the relative positions of the specific nested primers. Target products in the secondary reactions were slightly bigger than those in the tertiary reactions in accordance with the nested positions of the primers.

Types I and II PCR-based genome walking strategies described in the Introduction section require additional manipulations before PCR, such as restriction enzyme digestion followed by self-circularization or ligation of the adaptor to the target DNA fragments [1,9,11,12]. In the type II methods, amplification of undesirable products that are bound by the adaptor at both ends often results in high background levels [23]. For type III, some methods still require sample handling after amplification, such as exonuclease treatment and screening of target molecules [14,15]. TAIL-PCR and SFA-PCR are completely PCR-based; however, the amplified products of TAIL-PCR are usually small, and are subject to high background levels due to the use of short degenerate primers and a plurality of reduced-stringency cycles [1,14]. In SFA-PCR, target sequences cannot always be obtained [13].

Compared to the above methods, POP-PCR has one or more of the following merits: (1) Simplicity, POP-PCR does not need any DNA manipulation before PCR or laborious screening afterward, and the products can be directly sequenced using the PCR primers; (2) Specificity, as it specifically amplifies the desired DNA fragment while non-target products are eliminated by altering the combination of POP primer and specific primer; and (3) Efficiency, its efficiency is exemplified by the fact that 100% of the DNA walking experiments yielded a large size of specific products.

## Supporting Information

S1 Table Thermal cycling parameters used in the partially overlapping primer-based PCR method. (DOC)
