# Supplementary material for: Partially Overlapping Primer-Based PCR for Genome Walking
Source: PLoS One. 2015 Mar 26;10(3):e0120139. doi: 10.1371/journal.pone.0120139 (PMC4374871; doi:10.1371/journal.pone.0120139)
Supplement: S1 Table — (DOC) [file pone.0120139.s001.doc]

**S1 Table.** Thermal cycling parameters used in partially overlapping primer-based PCR method.

| Round of PCR | Stage | Thermal condition | Cycle number |
| --- | --- | --- | --- |
| Primary |  | 94℃ 1 min, 98℃ 1 min | 1 |
|  | 1 | 94℃ 30 s, 65℃ 1 min, 72℃ 2 min | 5 |
|  | 2 | 94℃ 30 s, 25℃ 1 min, 72℃ 2 min | 1 |
|  | 3 | 94℃ 30 s, 65℃ 1 min, 72℃ 2 min | 30 |
|  |  | 72℃ 5 min | 1 |
| 1 μL of the product was directly used as plate in the secondary round of PCR | | | |
| Secondary | 1 | 94℃ 30 s, 65℃ 1 min, 72℃ 2 min | 5 |
|  | 2 | 94℃ 30 s, 50℃ 1 min, 72℃ 2 min | 1 |
|  | 3 | 94℃ 30 s, 65℃ 1 min, 72℃ 2 min | 30 |
|  |  | 72℃ 5 min | 1 |
| 1 μL of the product was directly used as plate in the tertiary round of PCR | | | |
| Tertiary | Reaction profile of tertiary PCR is identical to that of secondary PCR | | |
